# Bolt-connected prefabricated cross-shaped I-steel tower crane foundation

**DOI:** 10.1371/journal.pone.0291982

**Published:** 2023-09-21

**Authors:** Jianhui Yang

**Affiliations:** Fuzhou Polytechnic, Fuzhou, Fujian, China; Covenant University, NIGERIA

## Abstract

Traditional tower cranes cannot meet the sustainable development goals as they use the cast-in-place concrete foundation, with large size, long construction period, and demolition after construction, resulting in waste of resources and high costs. This paper proposes a bolt-connected prefabricated cross-shaped I-steel tower crane foundation. It offers significant advantages in terms of convenient connection, low amortization cost and recyclability. The split connection point of the foundation is determined through the force analysis of the I-steel. With the ratio of the fixed cross-sectional area to the web height-to-thickness ratio (i.e. total cost) being constant, the inertia moment and bending stiffness of the I-steel are optimized using modern optimization design methods with the ratio of the web plate area to the total section area of the I-steel as the design variable, yielding the ideal strength and stiffness of the I-steel section.

## 1 Introduction

The use of cast-in-place concrete foundation by traditional tower cranes has problems such as large size, long construction cycle, and the tower crane can be installed with concrete of specified design strength after it is maintained for more than 7d following concrete pouring. Also, it is only for one-time use and costs a lot. Take the conventional QTZ40 tower crane foundation (4.5×4.5×1.2 m) as an example, its total costs, including construction costs (more than 30,000 yuan) and costs for demolition and garbage removal, reach up to 60,000 yuan, resulting in waste of resources and environmental pollution. To address the above-mentioned challenges, various forms of foundations were developed. For example, Zhao developed the prefabricated concrete tower crane foundation (Zhao Shi tower crane) [1]. Later, cross-shaped cast-in-place concrete foundations were developed based on different geological conditions. Su and Tan conducted a mechanical analysis of foundation beams [2]. Chen studied the theory and application of prefabricated cross-shaped steel tower crane foundation in detail [3]. After studying the basic construction technology of traditional fixed tower crane foundation, Jiang and Wan designed a 60T prefabricated cross-shaped steel tower crane foundation composed of cross-shaped steel, bottom steel plate, frame plate, etc., and verified its safety and reliability [4]. Hou et al. designed and calculated the square tower crane foundation with ANSYS three-dimensional analysis, VB reinforcement program and VB optimization program [5].

Based on the application status and existing problems of tower crane foundation, Ji put forward some ideas and structures for foundation design [6]. Sun conducted research on the structural design and mechanical properties of cross-shaped mobile tower crane [7]. Zuo carried out in-depth research on the design, positioning and construction of steel lattice column tower crane foundation [8]. Tan proposed a prefabricated steel structure tower crane foundation and conducted in-depth research [9]. Zhang et al. studied the construction technology of prestressed cross polygona assembled fiber concrete tower crane foundation, which is a multi-purpose combination and suitable for different forms of tower foundations [10]. The foundation can work repeatedly without installing, with low amortization cost, so it has certain engineering application value. To overcome the defects of cast-in-place concrete foundation, Fan et al. proposed to replace concrete foundation with prefabricated foundation, and introduced the advantages, construction technology and precautions of the prefabricated foundation [11]. Lei et al. made a detailed report on the basic technology of prefabricated prestressed concrete tower crane foundation at the 28 th East China Civil Engineering Construction Technology Exchange Conference, which greatly promotes the popularization and application of prefabricated foundation [12].

Each of the above foundations has advantages and dis advantages. For example, the prefabricated concrete tower crane foundation can save huge costs for engineering measures, compared with a traditional tower crane, but it requires high costs for transportation and lifting, with difficult assembly and disassembly by professional prestressed construction teams. The author, with years of engineering practice experience, proposes a bolt-connected prefabricated cross-shaped I-steel tower crane foundation. With convenient connection, high rigidity, low investment, short construction period, and recyclability, it has acquired utility model patent licensing for its good economic and social benefits. In this paper, the split connection point of the foundation is determined through the force analysis of the I-steel, and the section of the I-steel is optimized using modern optimization design methods, making the foundation achieve good performance with the total cost being constant.

## 2 Bolt-connected prefabricated cross-shaped I-steel tower crane foundation

### 2.1 Bolt connection type

**Fig 1:**
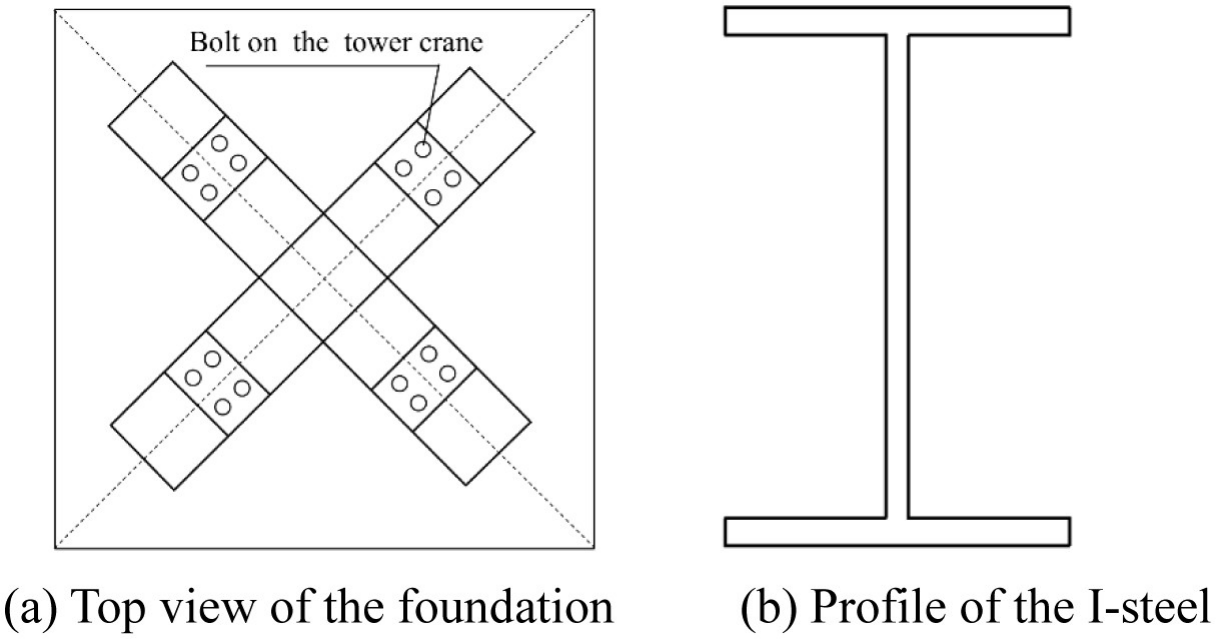
Bolt-connected prefabricated cross-shaped I-steel tower crane foundation. (a) Top view of the foundation. (b) Profile of the I-steel.

The bolt-connected prefabricated cross-shaped I-steel tower crane foundation is shown in Fig 1 below.

The length and width of tower crane foundation is generally more than 4 m, which does not meet the road transportation regulations in China. So we need to split one of the I-steels constituting the foundation into two or three parts, and then transfer them to the construction site for assembly. According to the principle of structural mechanics, the key parts of the I-steel, including the bending moment position and the maximum shear position such as the column base of tower crane, should be avoided when splitting. The specific split connection point can only be determined through mechanical analysis.

**Fig 2:**
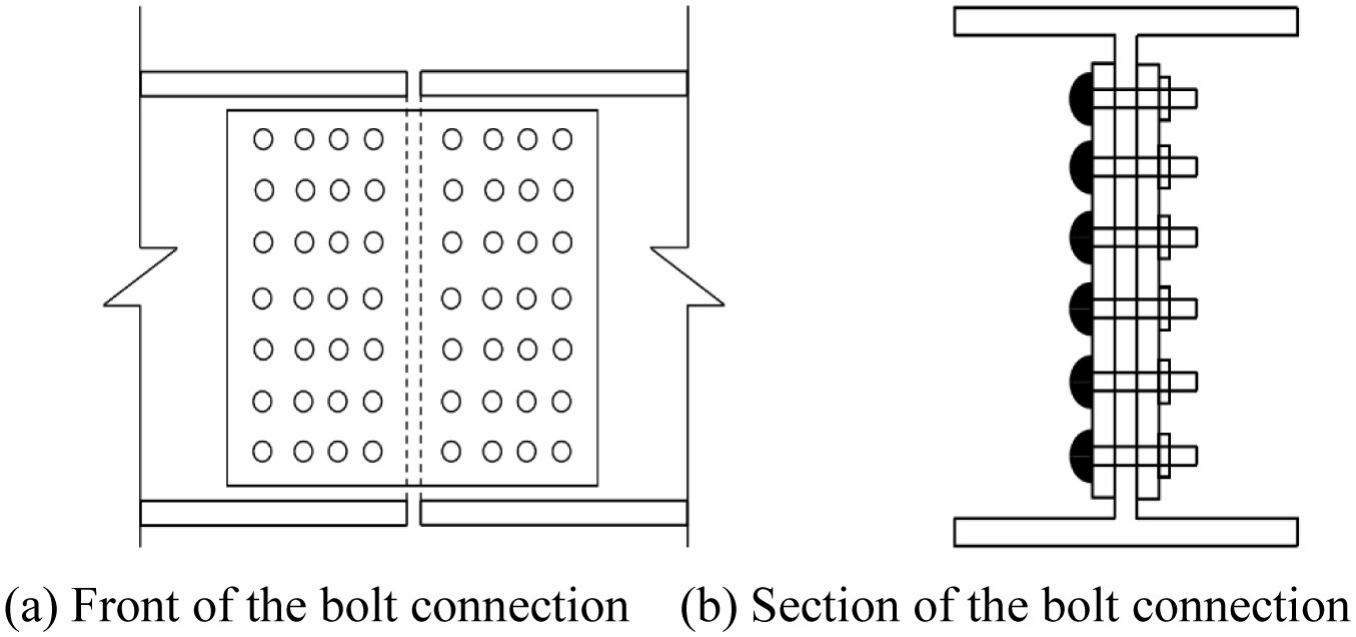
Architectural detail drawing of the bolt connection. (a) Front of the bolt connection. (b) Section of the bolt connection.

The bolt connection joint is shown in Fig 2 below. Both grades A, B and C ordinary bolts and high strength friction grid bolts (HSFG) meeting the shear and tensile strength of bolts and steel plates can be used. The bolt end distance and hole distance of bolt connection can be determined based on literature while meeting the requirements of Reference [13–15].

### 2.2 Foundation types for the foundation soils with different bearing capacity

**Fig 3:**
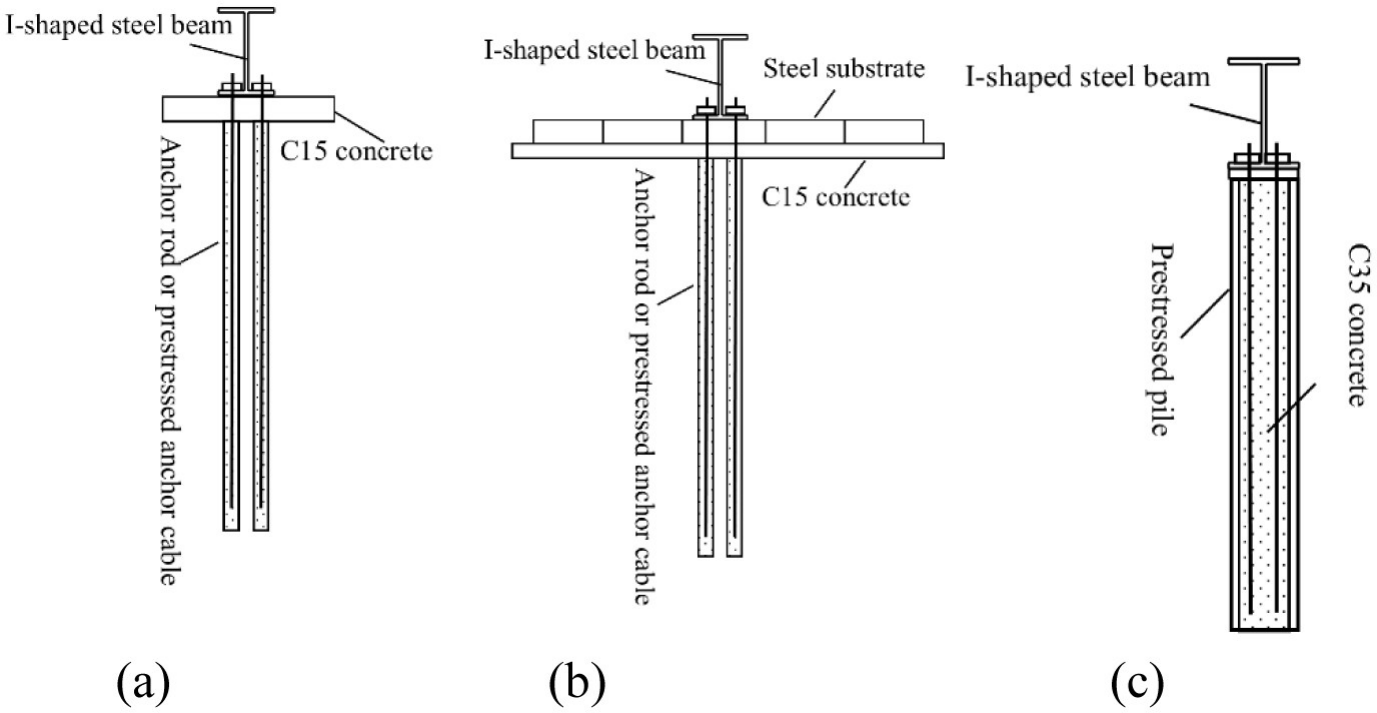
Foundation types for the foundation soils with different bearing capacity (a) Foundation soil with good bearing capacity (b) Foundation soil with general bearing capacity (c) Foundation soil with poor bearing capacity.

For the foundation soils with different bearing capacity, different foundation types are used:

For the foundation soil with good bearing capacity (generally ≥180 kPa), the I-steel is placed on C15 concrete that is first poured as a cushion layer, and then fixed with anchor rod or prestressed anchor cable to balance the overturning moment. The precast concrete blocks can also be stacked on the steel substrate to balance the overturning moment, as shown in Fig 3a. For the foundation soil with general bearing capacity, the steel substrate is placed on C15 concrete that is first poured as a cushion layer, and then fixed with anchor rod or prestressed anchor cable to balance the overturning moment, as shown in Fig 3b. For the foundation soil with poor bearing capacity, such as silt, the I-steel is placed on prestressed pile foundation that is first constructed, and the pile core is filled with C35 concrete to make anchor rod or prestressed anchor cable to balance the overturning moment, as shown in Fig 3c.

The foundation has the advantages of good integrity, large stiffness, non-cooperation of professional teams, convenient assembly and disassembly, convenient assembly and disassembly, and recyclability, producing good economic and social benefits.

## 3 Force analysis of the foundation

The force analysis of the cross-shaped I-steel is mainly to determine the split connection part of the foundation. Take the conventional QTZ40 tower crane foundation as an example. The basic calculation parameters are as follows: dead weight (including counterweight) 401.4 kN, maximum lifting load 60 kN, maximum load moment of tower crane 690 kNm, lifting height of tower crane 43 m, and width of tower crane 1.60 m. Provisional foundation length 5,000 mm, section size 400 × 1,000 mm (width × height), thickness of web plate and flange plate 20 mm, size of connecting plate 900 × 600 × 20 mm (the final size is determined according to the number of bolts after calculation and for economic reasons), and size of stiffener 150 × 600 × 20 mm (width × height × thickness, 1,000 mm / piece, double-sided layout).

**Fig 4:**
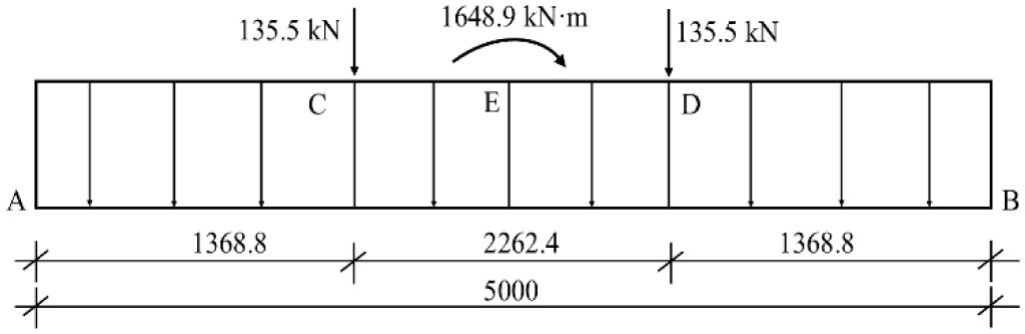
Structural compute diagram of the single I-steel.

The force form and characteristics of the foundation are very similar to those of cross-shaped strip foundation under column. According to Reference [16, 17], we split the cross-shaped I-steel into single steels for internal force calculation. The standard value of wind load in the diagonal direction of tower crane section is 0.2 kN/m^2^ in the in-service state, and 0.7 kN/m^2^ in the out-of-service state. The structural compute diagram of the single I-steel is shown in Fig 4 below, and C, D is the tower crane column base position. Detailed calculation process is omitted, and the lateral horizontal component force is not marked.

### 3.1 Bar system finite element method

The calculation results of Reference [2] may have deviations without considering the interaction between the foundation and the foundation soil, while the Winkler elastic foundation-based bar system finite element method can achieve high accuracy through simple and fast calculation [18–20]. The elastic modulus of foundation soil takes 11 MPa, the Poisson’s ratio takes 0.3, and the resistance coefficient of foundation soil is calculated according to *k*_*s*_ = *E*_s_[*b*(1-υ^2^)*I*_c_]^-1^, where *I*_c_ is the influence factor of foundation steel angle point. It is calculated to be 2.19 according to Table 1 [17], with the foundation assumed as rigid foundation. The elastic modulus of the spring of the foundation in this paper is determined according to the following formula:

Spring at the beam end: elastic foundation beam

$$E=\frac{kbl_{\text{d}}}{2}$$ (1)

Spring at other nodes:

$$E=kbl_{z}$$ (2)

Where *l*_d_ and *l*_z_ are the division length of bar system element at the beam end and middle respectively, and the section size and elastic parameters are the same as the aforementioned I-steel.

**Table 1:** Optimization results of welded I-steel section.

No	*α*	*I*/*I*_*utl*_	*W*/*W*_*utl*_
1	0.05	0.129	0.458
2	0.1	0.249	0.626
3	0.15	0.360	0.739
4	0.2	0.462	0.822
5	0.25	0.556	0.884
6	0.3	0.640	0.929
7	0.35	0.716	0.962
8	0.4	0.782	0.984
9	0.45	0.840	0.996
10	0.5	0.889	1.000
11	0.55	0.929	0.996
12	0.6	0.960	0.986
13	0.65	0.982	0.969
14	0.7	0.996	0.946
15	0.75	1.000	0.918
16	0.8	0.996	0.885
17	0.85	0.982	0.847
18	0.9	0.960	0.805
19	0.95	0.929	0.758
20	1	0.889	0.707

To ensure the calculation accuracy, the bar system strip foundation adopts the three-node quadratic space beam element, namely B32 element. The dimensions of each section are shown in Fig 3. The CD section is divided into two sections. The first section of AC section is divided by *l*_d_ = 0.3688 m, and the rest are divided by 0.2 m. Five sections of CE section are divided by 0.2 m, and the last section is divided by 0.1312 m. The ED and DB sections are divided in opposite dimensions. Thus, the strip I-steel is divided by non-equal length elements.

**Fig 5:**
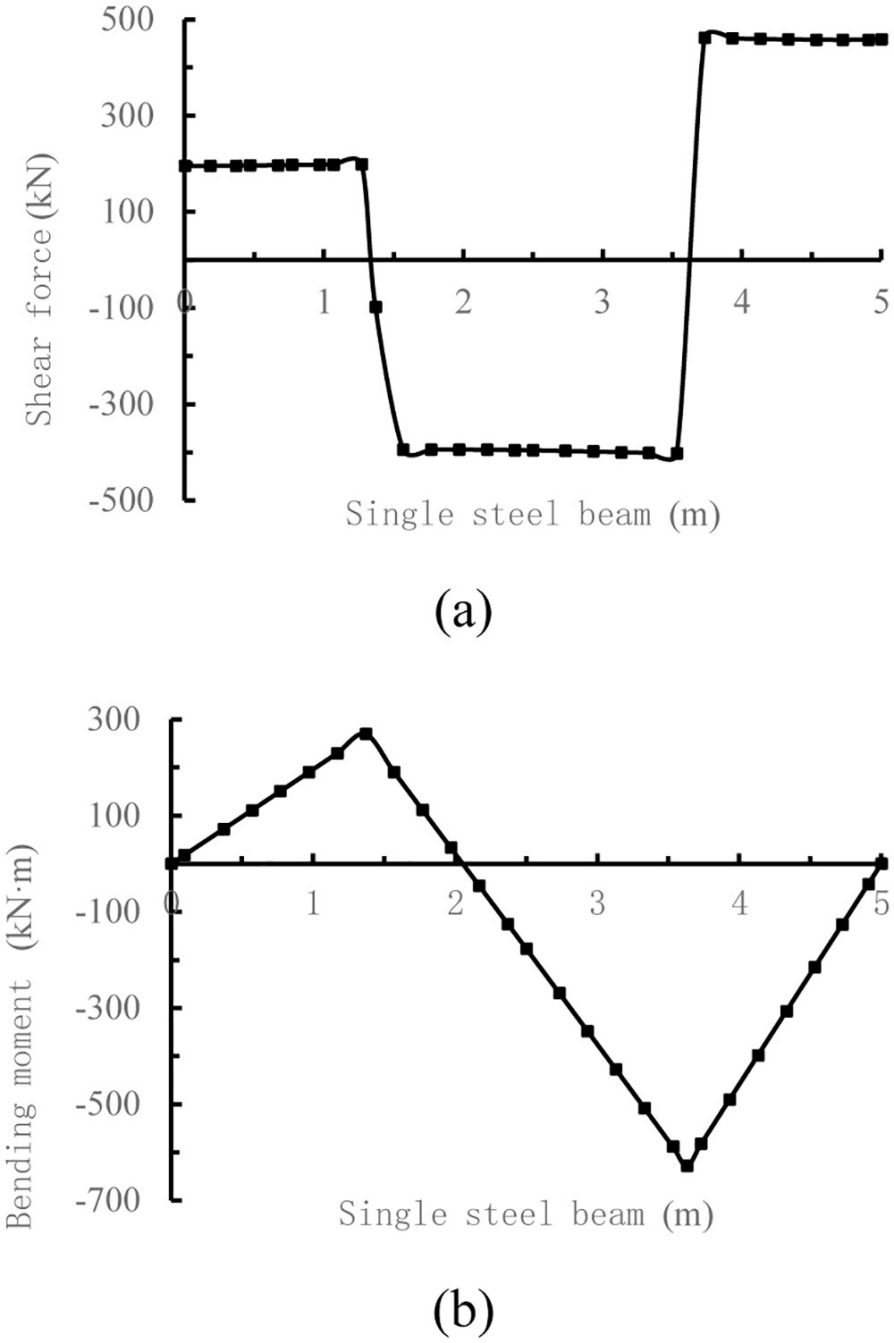
Internal force of the single I-steel by bar system finite element method. (a) Shear force of the single I-steel by bar system finite element method; (b) Bending moment of the single I-steel by bar system finite element method.

Simplified calculation is used to convert the concentrated couple of tower crane into a pair of concentrated forces *F*_*C*_ = 593.4 kN, *F*_*D*_ = 866.3 kN, as shown in Fig 5 below.

### 3.3 Three-dimensional solid finite element method

**Fig 6:**
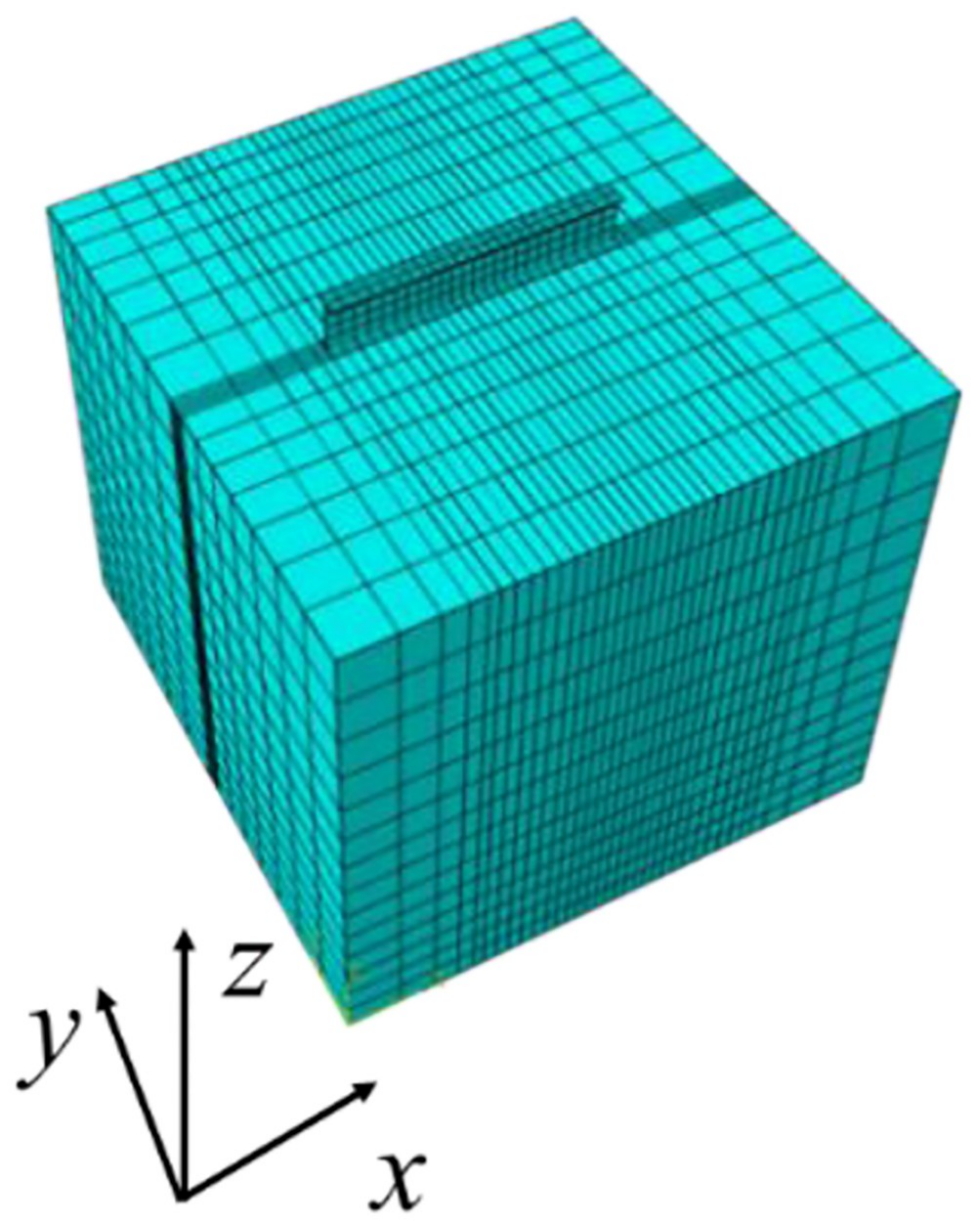
Three-dimensional solid finite element model and mesh division.

The three-dimensional solid finite element model is established with the parameters of section 2.1 above. The three-dimensional solid finite element model and mesh division are shown in Fig 6 below, and the dimension of the foundation soil model is 10×10×10 m.

**Fig 7:**
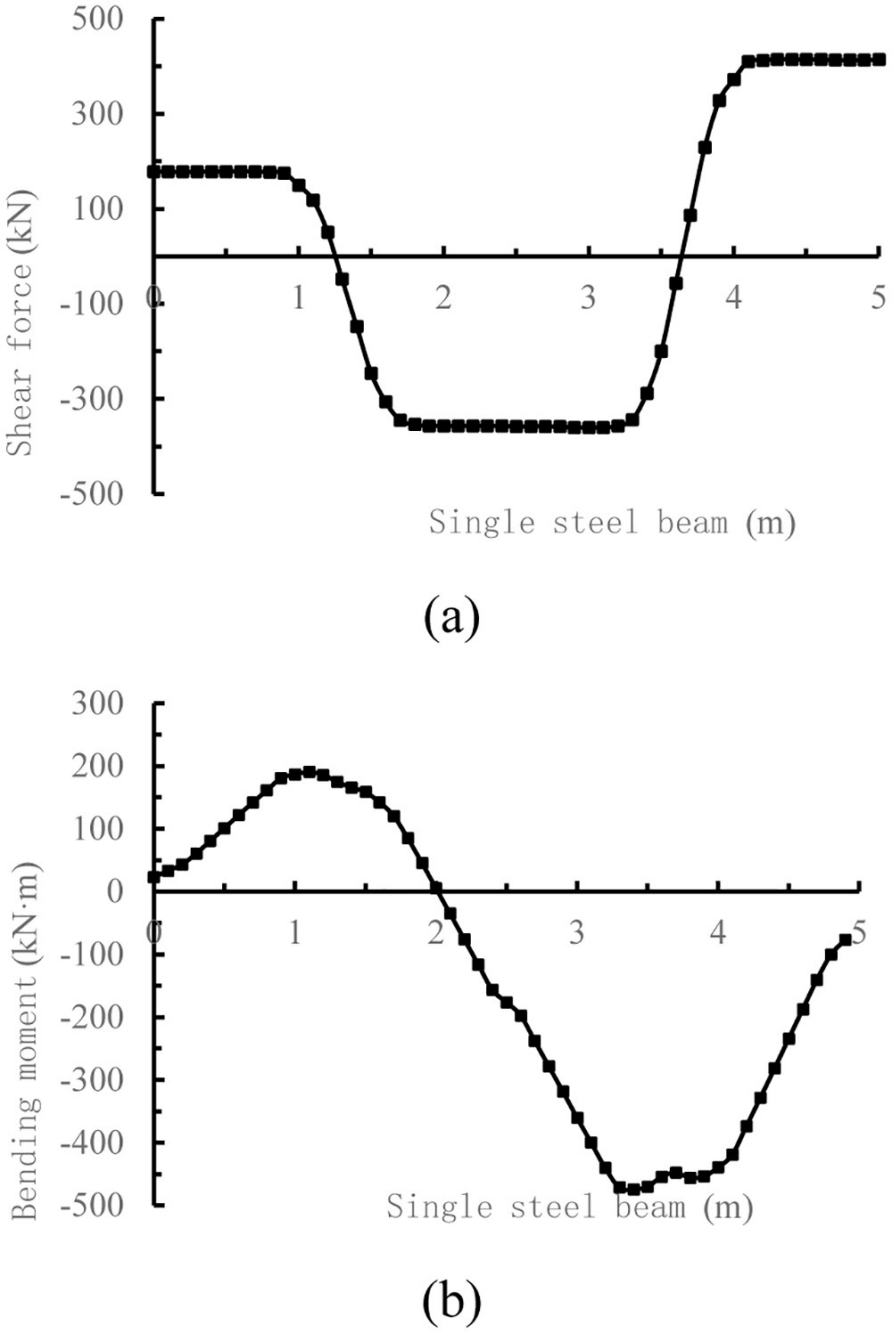
Internal force of the single I-steel by three-dimensional solid finite element. method (a) Shear force of the single I-steel by three-dimensional solid finite element method; b) Bending moment of the single I-steel by three-dimensional solid finite element method.

Suppose that the foundation soil and the I-steel are isotropic and homogeneous elastic materials, the first-order linear element C3D8I is used for the foundation soil and the single I-steel, and the concentrated forces (*F*_*C*_ = 593.4 kN and *F*_*D*_ = 866.3 kN respectively) of column base are converted to the average distributed loads of 3708.75 kPa and 5414.38 kPa respectively. In order to improve the calculation accuracy, the offset of ABAQUS is used to encrypt the mesh in the I-steel area. Boundary conditions constrain the x, y, z direction displacement of the foundation soil model and constrain the x-direction displacement of the I-steel ends. The internal force of I-steel is extracted by slicing at 0.1 m spacing, as shown in Fig 7 below.

### 3.4 Comparative analysis of calculation results

**Fig 8:**
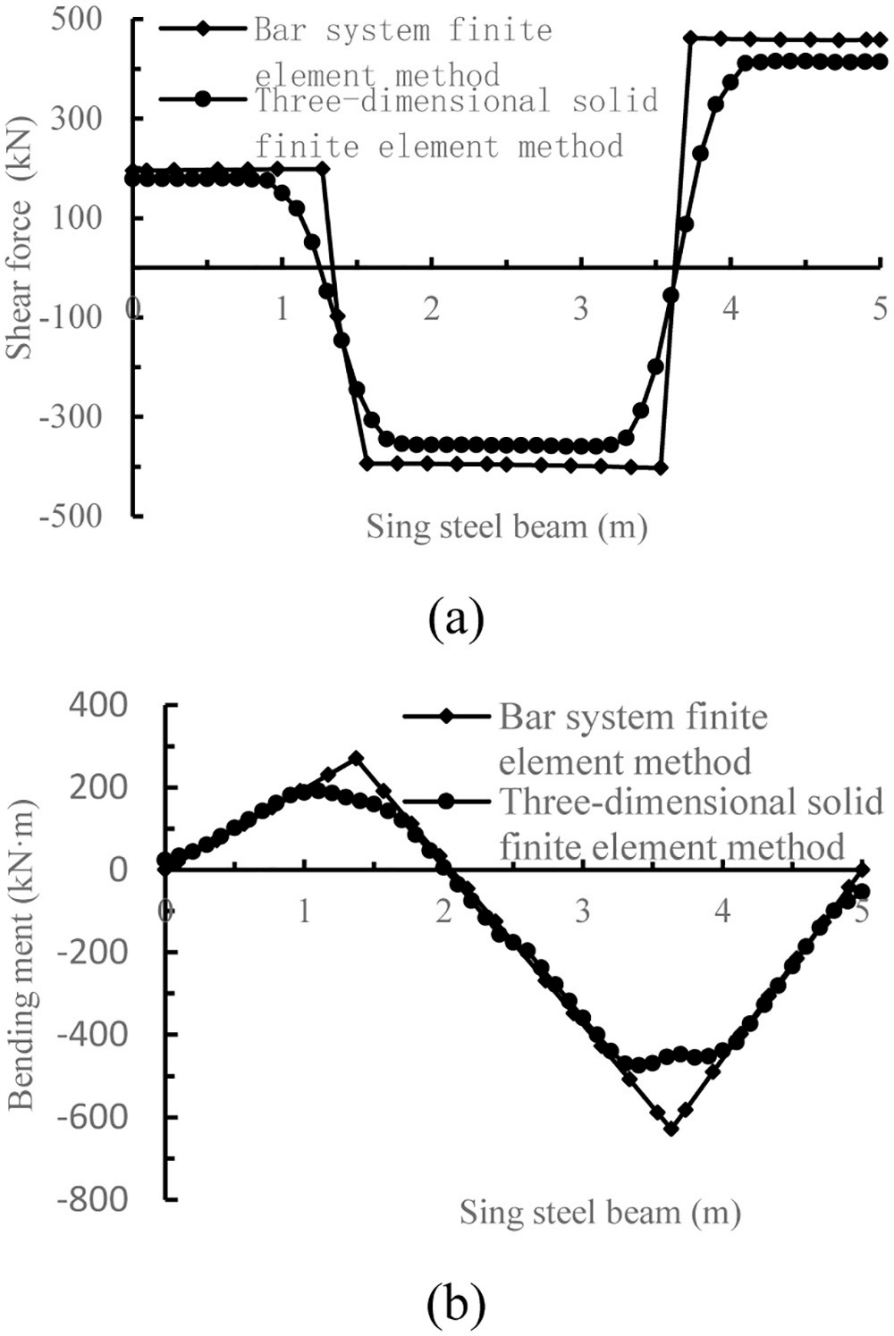
Comparison of internal force of the single I-steel by three different methods. (a) Comparison of shear force of the single I-steel by three different methods; (b) Comparison of bending moment of the single I-steel by three different methods.

The calculation results of Figs 5 and 7 are compared, as shown in Fig 8.

From the comparison diagram of internal force distribution in Fig 8, it can be seen that the internal force distribution form of the bar system finite element method and the three-dimensional solid finite element method is basically the same, but the peak shear force of the bar system finite element method is 45.8 kN larger than that of the three-dimensional solid finite element method, and the peak bending moment of the bar system finite element method is 154 kN·m larger than that of the three-dimensional solid finite element method. Thus, there are large deviations in the calculation results of the two methods, which remains to be verified by follow-up studies.

At the same time, it can be seen from Fig 8 that the internal force at the cross joint of the I-steel is relatively small, and it is more reasonable to choose this place as the split point of the I-steel. After the internal force distribution, size and split connection point of the I-steel are determined, the weld, bolt number and stiffener configuration design can be carried out according to Reference [10], which will not be described in detail here.

## 4 Optimization design

The cross-shaped I-steel is the main stress component of the foundation in this paper, and its design quality is directly related to the safety and economy of the tower crane. This section, with the ratio of the web plate area to the total section area of the I-steel as the design variable, intends to combine the optimization design of bridge crane and gantry crane steel [17, 21] as well as engineering optimization method [22–30] to optimize the moment of inertia (*I*) and bending modulus (*W*) of the I-steel under the constraints of total steel plate consumption and height-to-thickness ratio of web plate to seek the ideal strength and stiffness of the I-steel section, making the foundation achieve good performance with the total cost being constant.

(1) Dimensions and parameters of the I-steel section

**Fig 9:**
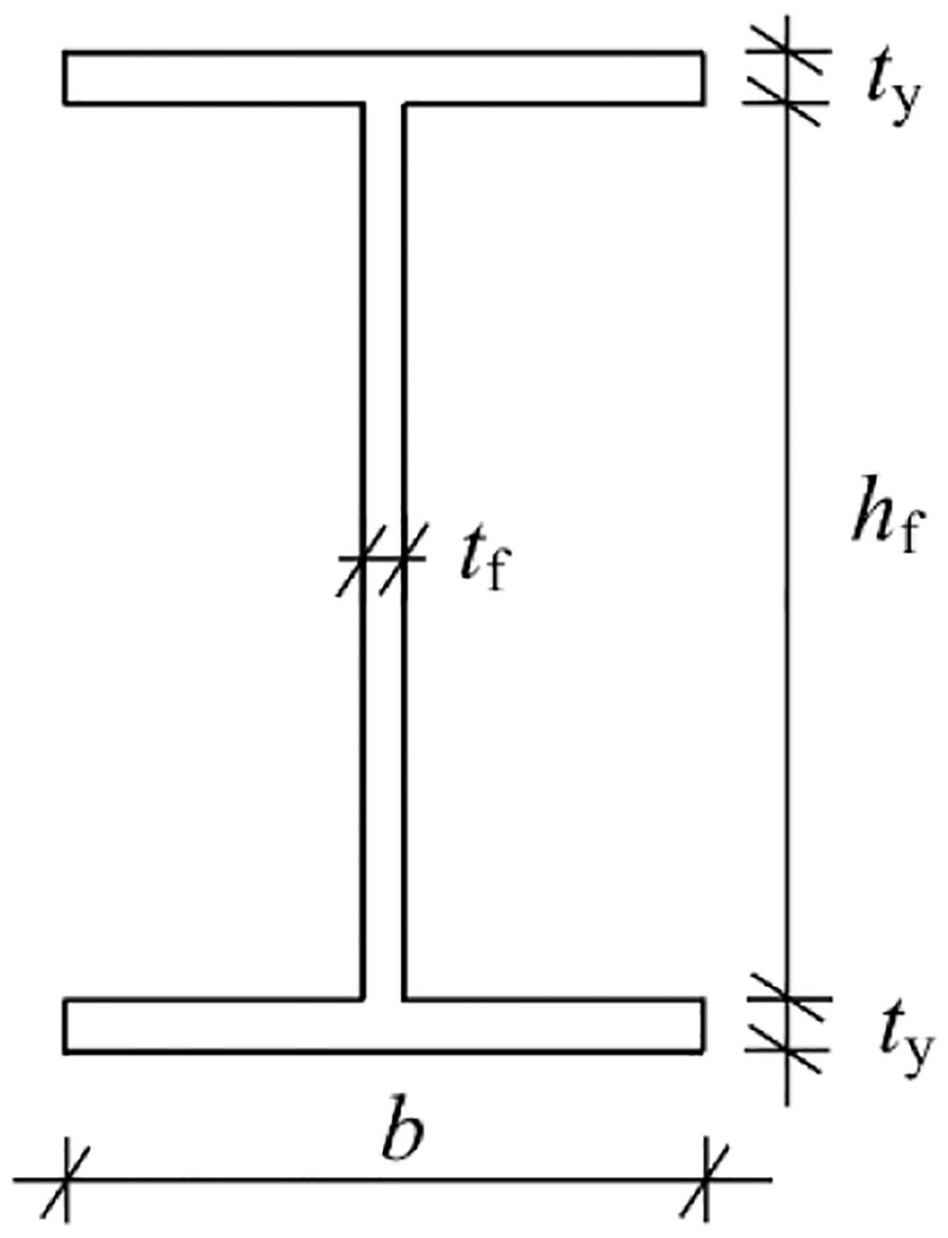
Section of the I-steel.

The dimensions and parameters of the I-steel section are shown in Fig 9 below.

Flange plate area of the I-steel:

$$S_{\text{y}}=2bt_{\text{y}}$$ (3)

Web plate area of the I-steel:

$$S_{\text{f}}=h_{\text{f}}t_{\text{f}}$$ (4)

Total section area of the I-steel:

$$S=S_{\text{y}}+S_{\text{f}}=2bt_{\text{y}}+h_{\text{f}}t_{\text{f}}$$ (5)

(2) Function expression of the moment of inertia *(I)* and bending modulus (*W*) of the I-steel section

To simplify the calculation, assume that the top and bottom flange plates of the I-steel are concentrated on the neutral wire. Let *α* be the ratio of the web plate area and the total section area, *α* = *S*_f_/*S*; *β* is the height-to-thickness ratio of the web plate, *β* = *h*_f_/*t*_f_. Thus, *I* and *W* are respectively:


β=hf/tf

$$\begin{array}{cc} I & =\frac{t_{\text{f}}h_{\text{f}}{}^{3}}{12}{\text{+S}}_{\text{y}}{\left(\frac{h_{\text{f}}}{2}\right)}^{2}=\frac{t_{\text{f}}h_{\text{f}}h_{\text{f}}{}^{2}}{12}\text{+}\frac{\left(\text{S-}h_{\text{f}}t_{\text{f}}\right)h_{\text{f}}{}^{2}}{4}=\frac{\alpha Sh_{\text{f}}{}^{2}+3\left(S-\alpha S\right)h_{\text{f}}{}^{2}}{12}\\ & =\frac{\alpha Sh_{\text{f}}{}^{2}+3Sh_{\text{f}}{}^{2}-3\alpha Sh_{\text{f}}{}^{2}}{12}=\frac{\left(3-2\alpha \right)Sh_{\text{f}}{}^{2}}{12} \end{array}$$ (6)

We obtain *h*_f_ = *αS*/*t*_f_ from *α* = *S*_f_/*S* = *h*_f_*t*_f_/*S* and *h*_f_ = *βt*_f_ from *β* = *h*_f_/*t*_f_, then, $h_{\text{f}}{}^{2}=h_{\text{f}}h_{\text{f}}=\alpha S/t_{\text{f}}\times \beta t_{\text{f}}=S\alpha \beta$. By substituting it into Eq (6) we obtain:

$$I=\frac{\left(3-2\alpha \right)\alpha \beta S^{2}}{12}$$ (7)

W=Ihf/2=2Ihf=3−2ααβS26αβS=3−2αα12β12S326 (8)

(3) Optimization of the moment of inertia *(I)* and bending modulus (*W*) of the I-steel section

Section design is performed after the external load of the foundation is determined through calculation. The optimization design principle is to have the minimum *S* with the lowest cost of the I-steel per unit length under the constraints of strength, stiffness and stability. At the same time, the optimal web plate height can allow *I* and *W* to reach the extremum.

With *α* as the design variable, we find the derivative of Eqs (7) and (8) when *S* and *β* are the fixed values. Then we obtain:

$$\frac{dI}{d\alpha }=\frac{1}{4}\beta S^{2}-\frac{1}{3}\beta S^{2}\alpha$$ (9)

$$\frac{dW}{d\alpha }=\frac{\beta^{\frac{1}{2}}S^{\frac{3}{2}}}{4\alpha^{\frac{1}{2}}}-\frac{\alpha^{\frac{1}{2}}\beta^{\frac{1}{2}}S^{\frac{3}{2}}}{2}$$ (10)

Let $\frac{dI}{d\alpha }=0$, that is, when $\alpha_{\text{utl}}=\frac{3}{4}$,

$$I_{\text{utl}}=\frac{\left(3-2\times 3/4\right)\times 3\times \beta S^{2}}{12\times 4}=\frac{3\beta S^{2}}{32}=\frac{h_{\text{utl}}{}^{4}}{6\beta }$$ (11)

Let $\frac{dW}{d\alpha }=0$, that is, when $\alpha_{\text{utl}}=\frac{1}{2}$,

$$W_{\text{utl}}=\frac{\left(3-2\times \frac{1}{2}\right){\left(\frac{1}{2}\beta \right)}^{\frac{1}{2}}S^{\frac{3}{2}}}{6}=\frac{\sqrt{2}\beta^{\frac{1}{2}}S^{\frac{3}{2}}}{6}=\frac{2h_{\text{utl}}{}^{3}}{3\beta }$$ (12)

Eqs (11) and (12) show that when *S* is certain, there is a clear *β* to make the stiffness of the I-steel optimal (with *h*_f_/ *t*_f_ and stiffener to ensure the local stability of welded I-steel). When *S*_f_/*S* = 3/4, the stiffness of the I-steel is optimal, that is, the I-steel has the optimal resistance to bending; when *S*_f_/*S* = 1/2, the bending modulus of the I-steel is the largest, the material strength is the best used, and the optimization solution of the I-steel height can be obtained.

By dividing Eqs (7) and (8) by Eqs (11) and (12) respectively we obtain:

$$\frac{I}{I_{utl}}=\frac{8\left(3-2\alpha \right)\alpha }{9}$$ (13)

$$\frac{W}{W_{utl}}=\frac{\sqrt{2}\left(3-2\alpha \right)\alpha^{\frac{1}{2}}}{2}$$ (14)

For *α*, we take values of 0 and 1. Then we obtain Table 1 by Eqs (13) and (14).

**Fig 10:**
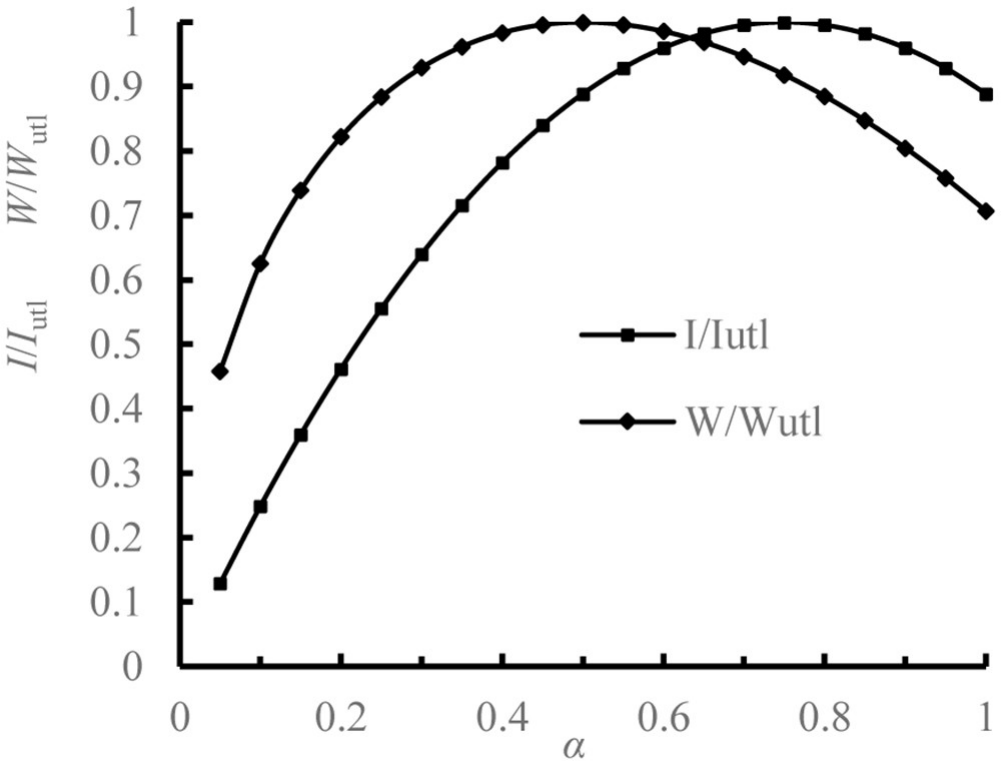
Relation curves between *α* and *I*/*I*_*utl*_ and *W*/*W*_*utl*._

We draw the optimization results of Table 1 and obtain the relation between *α* and *I*/*I*_*utl*_ and *W*/*W*_*utl*_, as shown in Fig 10 below.

From Table 1 and Fig 10 we conclude:

1. *I* and *W* increase first and then decrease with the increase of *α*, and they have an extremum. When *α* = 0.75, *I* reaches the optimal value; when *α* = 0.5, *W* reaches the optimal value.
2. When *α* = 0.65~0.85, *I* is within 98% of the optimal value; when *α* = 0.4~0.6, *W* is within 98% of the optimal value.
3. When *α* = 0.631, the two curves intersect, both *I* and *W* are within 97.4% of the optimal value.

Thus, the appropriate *α* can be selected according to the external load of the I-steel, and then *I* and *W* are solved with minimum *S* and determined *β*, and then *α* is adjusted according to specification requirements. At the same time, *I* and *W* are guaranteed to meet requirements.

According to *I* = *M/*(*Ey*) in mechanics of materials, the minimum *I* can be calculated after determining the bending moment (*M*) and the allowable deflection (*y*) of the I-steel. Take the above simplified calculation parameters as an example, *M* = 669.1 kN·m, *y* = *l*/500 = 10 mm, then $I\geq \frac{M}{Ey}=\frac{669.1}{2.06\times 10^{8}\times 0.01}=3.25\times 10^{8}{\text{mm}}^{\text{4}}$.

According to Zuo [8], the stability of the flange plate and the web plate should be considered to avoid local instability and affect the strength, stiffness and overall stability of the I-steel. The local stability of flange plate requires that there is no support of compression flange plate between the web plates, and the width-to-thickness ratio does not exceed 40 *ε*_k_. The local stability of the web plate is related to the force, height-to-thickness ratio and properties of the material of the web plate. Under the action of local compressive stress, shear stress and bending stress, the height-to-thickness ratio of the web plate should be lower than 84 *ε*_k_, 104 *ε*_k_ and 174 *ε*_k_ respectively. Where *ε*_k_ is the correction coefficient of steel grade, which is the square root of the ratio of 235 to the yield point value in the steel grade.

According to the above requirements, the foundation takes *β* = 100, and *h*_utl_ = 664 mm is obtained from Eq (11) and it takes *h*_utl_ = 700 mm. For *t*_f_ = *h*_utl_/*β* = 7 mm, let *t*_f_ = 10 mm, then the area of web plate is *S*_f_ = 7,000 mm^2^.

Section stiffness is checked by substituting the above parameters. The *W* of the I-steel section (700×250×10×10 mm) is 8.57×10^8^ mm^4^, which is greater than 3.25×10^8^ mm^4^ required by the internal force calculation of the I-steel, so it meets the stiffness requirements.

## 5 Conclusion

To overcome the defects of the traditional cast-in-place concrete tower crane foundation, the bolt-connected prefabricated cross-shaped I-steel tower crane foundation is proposed. By comparing the mechanical calculation results of different methods, it is determined that the cross joint of the I-steel is the appropriate split connection point. Both grades A, B and C ordinary bolts and high strength friction grid bolts (HSFG) meeting the shear and tensile strength of bolts and steel plates can be used for bolt connection. The foundation has the advantages of convenient assembly and disassembly, and recyclability, producing good economic and social benefits.

The section of the I-steel is optimized using modern optimization design methods. When the ratio of the web plate area to the total section area of the I-steel is 0.631, the foundation has good stiffness and strength with the total cost being constant.
